# Herbaceous Legume Encroachment Reduces Grass Productivity and Density in Arid Rangelands

**DOI:** 10.1371/journal.pone.0166743

**Published:** 2016-11-17

**Authors:** Thomas C. Wagner, Susanne Hane, Dave F. Joubert, Christina Fischer

**Affiliations:** 1 Restoration Ecology, Department of Ecology and Ecosystem Management, Technische Universität München, Freising, Germany; 2 Institute for Physical Geography and Landscape Ecology, Leibniz Universität Hannover, Hannover, Germany; 3 Natural Resources and Spatial Sciences, Namibia University of Science and Technology, Windhoek, Namibia; Technion Israel Institute of Technology, ISRAEL

## Abstract

Worldwide savannas and arid grasslands are mainly used for livestock grazing, providing livelihood to over a billion people. While normally dominated by perennial C_4_ grasses, these rangelands are increasingly affected by the massive spread of native, mainly woody legumes. The consequences are often a repression of grass cover and productivity, leading to a reduced carrying capacity. While such encroachment by woody plants has been extensively researched, studies on similar processes involving herbaceous species are rare. We studied the impact of a sustained and massive spread of the native herbaceous legume *Crotalaria podocarpa* in Namibia’s escarpment region on the locally dominant fodder grasses *Stipagrostis ciliata* and *Stipagrostis uniplumis*. We measured tussock densities, biomass production of individual tussocks and tussock dormancy state of *Stipagrostis* on ten 10 m x 10 m plots affected and ten similarly-sized plots unaffected by *C*. *podocarpa* over eight consecutive years and under different seasonal rainfalls and estimated the potential relative productivity of the land. We found the percentage of active *Stipagrostis* tussocks and the biomass production of individual tussocks to increase asymptotically with higher seasonal rainfall reaching a maximum around 300 mm while the land’s relative productivity under average local rainfall conditions reached only 40% of its potential. *Crotalaria podocarpa* encroachment had no effect on the proportion of productive grass tussocks, but reduced he productivity of individual *Stipagrostis* tussocks by a third. This effect of *C*. *podocarpa* on grass productivity was immediate and direct and was not compensated for by above-average rainfall. Besides this immediate effect, over time, the density of grass tussocks declined by more than 50% in areas encroached by *C*. *podocarpa* further and lastingly reducing the lands carrying capacity. The effects of *C*. *podocarpa* on grass productivity hereby resemble those of woody encroachers. Therefore, against the background of global change, the spread of herbaceous legumes and the underlying patterns needs to be further investigated to develop adequate counter measures for a sustainable land use.

## Introduction

The savannas and grasslands of arid and semi-arid drylands in the subtropics and tropics make up more than 20% of the earth’s land surface. Dominated by often protein-rich perennial C_4_ grasses, whose stalks and panicles supply fodder for wildlife and livestock, more than 70% of these lands are used for pasture and provide livelihood to over a billion people [1]. An increasing number of these rangelands are affected by the recent and massive proliferation of C_3_ species [2,3]. These encroachers are typically native woody, perennial legumes but occasionally herbaceous plants or invasive alien species [4–6], that lastingly change the community structure and repress the formerly dominant grasses [7,8]. The drivers of such dominance shifts are manifold [9–11] and include land-use changes such as unsustainable grazing practice [12,13], altered fire regime [14,15] but also climatic factors such as rainfall regime [12,13] or elevated atmospheric CO_2_ levels [2,16–18]. The ecological consequences of these encroachment processes are similarly diverse, and often ambiguous, ranging from negative through neutral to positive depending on the respective ecosystem and function [9,19]. However, there is a wide consensus that encroachment is associated with a loss of pasturage in the affected areas [9,20,21], leading to substantial declines of pastoral production and endangering local people’s livelihood. In Namibia more than 26 M ha are affected by encroachment of various woody legumes, such as *Senegalia mellifera* (Vahl), *Vachellia reficiens* (Wawra) or *Dichrostachys cinerea* (L.) Wight & Arn [22]. As over 60% of the agricultural production in Namibia originates from livestock farming [23] the annual losses are estimated to amount to over 70 M US$ [22], and similar numbers apply for South Africa [24] or Uganda [25]. This negative effect of encroachment on pastoral production has so far mainly been attributed to the encroachers’ spatial demands [21,26]. Studies demonstrating a direct (competitive) impact of the encroaching species on the grasses density and individual productivity are rare [27–29]. However, encroachment is not restricted to woody species and similar processes have been described for annual and perennial forbs and grasses [4,30,31]. Just like woody encroachers, these forbs or grasses exhibit massive increase in population densities, and, while themselves being unpalatable, reduce the pastures forage quality [4,22]. Since 2008 the herbaceous legume *C*. *podocarpa* has exhibited a massive spread in Namibia’s escarpment region between Namibrand in the South and Karibib in the North, leading to articulate complaints of local farmers about the plants’ negative impact on pasturing (T. C. Wagner, personal communication). With our study, we characterize and quantify the direct and long-term impact of this spread of *C*. *podocarpa* on the productivity of *Stipagrostis* grassland and the role of seasonal rainfall in grass biomass production between 2009 and 2016. We ask the following question: How does encroachment by *C*. *podocarpa* affect dormancy state, individual tussock productivity and tussock density of *Stipagrostis* under natural rainfall conditions?

## Material and Methods

### Study Area

The study was carried out on long term observation plots on the 7000 ha sized farm Rooiklip (S 23°24’23.29”, E 016°03’37.35”), which is situated in Namibia’s lower escarpment, a steep decline that constitutes a narrow transition zone between the more humid Nama-Karoo biome of the Namibian highveld and the Namib desert. The climate is hot-arid, and mean annual precipitation is 120 mm. Rainfall is generally erratic and predominantly occurs between October and March, with a pronounced high in February and March that defines the main growing season. The vegetation is dominated by a sparse matrix of the perennial tussock forming C_4_ grasses *Stipagrostis ciliata* and *Stipagrostis uniplumis* with an average density of 2 tussocks/m^2^ and a rainfall-dependent total canopy cover reaching a maximum of 50%. Interspersed are occasional trees, mainly *Senegalia reficiens* (formerly *Acacia reficiens*) or *Commiphora* sp. and shrubs such as *Orthanthera albida*, *Blepharis* sp. or *Petalidium* sp. During the rainy season, these perennials are complemented by annual grasses and various therophytes, among them the herbaceous legume *C*. *podocarpa*. As the annual plants are short-lived and rarely live longer than a few weeks, the main pasturage comprises the nutrient-rich perennial *Stipagrostis* species. Due to climate and vegetation, livelihood is primarily reliant on extensive livestock-keeping with goats and sheep, and, where rainfall and grass density suffice, cattle too. The farm Rooiklip was last used for farming at a larger extent in the 1970s. In 1981 the southern part of the farm was proclaimed a private game reserve, while the northern part of the farm is still used for extensive grazing with a total of 200–600 sheep. Our research on Rooiklip was approved by the Ministry of Environment and Tourism of Namibia, (research permit 1982/2014) and conducted with the explicit permission of the farm owners.

### Study Species

*Stipagrostis ciliata* (Desf.) De Winter and the closely related *Stipagrostis uniplumis* (Licht.) De Winter are perennial tufted C_4_-grasses that are widespread on sandy and stony soils in southern Africa where the mean annual precipitation ranges between 100 and 300 mm [32]. These grasses constitute the primary source of forage for livestock and native herbivores in the study area [33,34], their persisting dried up culms providing fodder long into the dry season. Both species are highly drought-tolerant and can remain dormant for up to two years under dry conditions. Under favourable conditions, tussocks can reach an age of seven years or more [35]. *Stipagrostis* propagates both vegetatively by means of rhizomes and reproductively through seeds. Their biomass production varies considerably with rainfall [36,37] while the general tussock density in the respective area corresponds with long-term average precipitation.

The annual, herbaceous legume *Crotalaria podocarpa* DC is common in arid parts of southern Africa [38], where it prefers sandy and stony soils. A symbiosis with *Methylobacterium nodulans* [39] allows nitrogen fixation and supports the species’ success in its nutrient-limited environments, as long as sufficient water is available. Its growth, number of flowers and seed set vary considerably with rainfall and water availability (T.C. Wagner and C. Fischer, unpublished data). Seeds are produced in high numbers, are viable for over four years and build up a persistent soil seed bank [40]. Due to its content of pyrrolizidine alkaloids and flavonoids, *C*. *podocarpa* is unpalatable for livestock [41,42]. In the study area, *C*. *podocarpa* used to occur in moderate numbers with less than 5 individuals per 100 m^2^ as an inconspicuous therophyte of the local grassland communities. It shares several significant life history traits with encroaching woody legumes in the region, such as *Vachellia* sp., *Senegalia* sp. or *Acacia* sp. (Table 1). Since 2008, according to complaints by local farmers and our personal observation, *C*. *podocarpa* has exhibited a pronounced and steady increase in the study region and since then even replaced *Stipagrostis* as the dominant species in some places during the growth period.

**Table 1:** Life-history traits of woody encroaching species and *C*. *podocarpa*.

Life-history trait	Encroaching woody legumes in southern Africa	*C*. *podocarpa*	Shared trait
Growth forms	shrub, tree	forb	no
Longevity	perennial	annual	no
Rooting pattern	tap root	tap root	yes
Protection against mammalian herbivores	yes (often thorns and/or poison)	yes (poison)	yes
Dry season survival	yes, dormancy	yes, seed	yes
Persistence	yes, phanerophyte	yes, seed bank	yes
Reproduction	generative	generative	yes
Dispersal mode	zoochorous	autochorous	no
Seed bank	transient	persistent	no
Photosynthetic pathway	C3	C3	yes
N-fixation ability	yes	yes	yes
**Shared traits**			**63%**

### Grass density and biomass production in relation to seasonal rainfall

For the selection of our study sites, a stratified random approach was taken. In 2009, two areas within the ungrazed southern part of the Rooiklip farm were selected one already encroached with *C*. *podocarpa* (initial total *C*. *podocarpa* cover ≥ 10%, later referred to as “affected sites”) and one with still normal *C*. *podocarpa* densities (initial total *C*. *podocarpa* cover ≤ 1%, later referred to as “unaffected sites”). Due to the high spatial variability of rainfall, both sites were located within a 2 km radius to ensure equal rainfall conditions. Within each area 10 plots of 10 m x 10 m were randomly selected with a minimum distance of 50 m (center-center) from each other and set up as long-term observation plots.

While already differing in *C*. *podocarpa* density, both areas initially had a similar density of *Stipagrostis* tussocks (2.12 ± 0.14 and 2.10 ± 0.10 tussocks/m^2^), and did not differ in soil types, soil texture and general vegetation structure (Table A in S1 Table) and rainfall. Rainfall data were recorded on a daily basis using a standard rain gauge, midway between affected and unaffected sites. For further analysis only rainfall occurring during the main rainy season and respective growth period between February and March was considered.

Between 2009 and 2016 the number of individuals, total cover and the area covered per individual plant (in m^2^) were determined for *Stipagrostis* and *C*. *podocarpa* at the end of the growing season in April each year before the die off of *C*. *podocarpa*. Cover of *Stipagrostis* tussocks was measured including dried up culms. *Stipagrostis* tussocks were counted and partitioned into active tussocks (with visible green blades) and inactive tussocks, the latter group consisting of dormant or actually dead tussocks, that did not produce new biomass during the current growing season. *C*. *podocarpa* individuals were included if at least one pinna was developed. No data were gathered in 2010 due to logistical problems. Individual cover was used as proxy for biomass production [37,43]. These data were used to model the effect of *C*. *podocarpa* on grass productivity and compare biomass production by individual grass tussocks on affected and unaffected sites under the respective seasonal rainfall conditions.

### Statistical Analysis

*Stipagrostis* tussock density on affected and unaffected sites was modelled over time using a linear mixed effect model with *year*, *rainfall* and their interaction as explanatory variables and manually simplified by eliminating insignificant factors *rainfall* and its interaction with *year*.

The percentage of active *Stipagrostis* tussocks in response to seasonal rainfall was modelled using a non-linear mixed effect model assuming asymptotic function through the origin (*SSasympOrig*: Asym*(1-exp(-exp(*lrc*)*input)), while the individual cover of *Stipagrostis* tussocks in relation to seasonal rainfall was modelled assuming an asymptotic function (*SSasymp*: *Asym*+(*R0*-*Asym*)*exp(-exp(*lrc*)*input)). The latter model was used to determine the lower threshold of rainfall necessary to support growth of *Stipagrostis* and the amount of precipitation at which grass productivity reaches 95% of the asymptotic maximum. The predicted values for the percentage of active tussocks and individual tussock cover were used to calculate the potential relative productivity in relation to seasonal rainfall (% active tussocks * predicted tussock area/predicted maximum tussock area) assuming a constant tussock density. The results were used to derive rainfall thresholds for 95% and 75% productivity and estimate relative grass productivity at the mean seasonal rainfall in the study area. Statistical analysis was performed with R 3.2.1 [44]. Linear and non-linear mixed effect models were implemented with *lme* or *nlme* (library *nlme* R package version 3.1–128, [45]) with maximum log likelihood and using ‘Plot’ as random factor to ensure independence of errors with respect to temporal autocorrelations [46]. Where possible, models were manually simplified by stepwise of non-significant factors until the minimal adequate model was reached.

## Results

**Fig 1:**
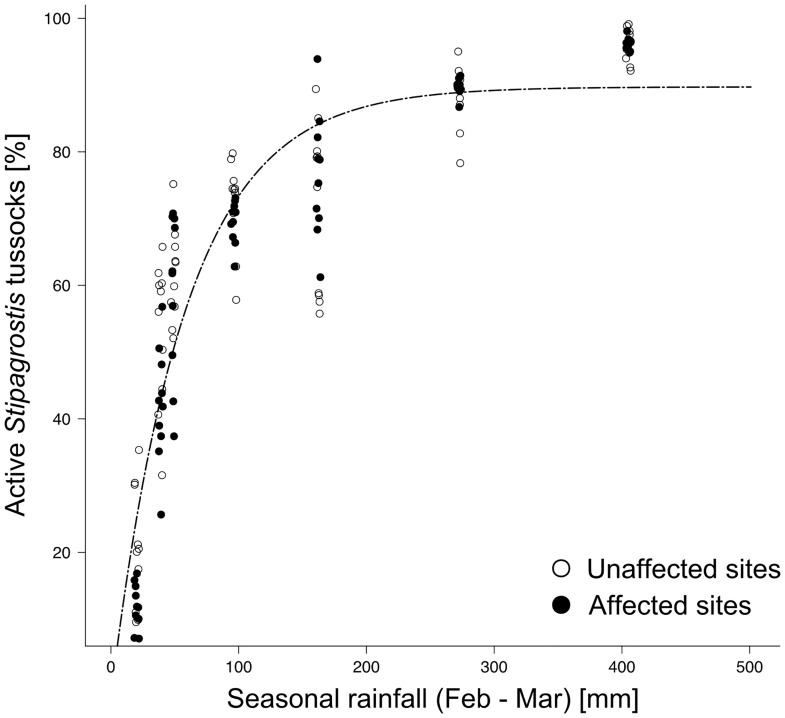
Percentage of active *Stipagrostis* tussocks (tussocks that produced biomass during the current growing season) in relation to seasonal rainfall (February-March) between 2009 and 2016. The predicted line from the non-linear mixed effect model is shown.

The percentage of active *Stipagrostis* tussocks was not different on affected and unaffected sites and increased asymptotically with seasonal rainfall with a logarithmic rate constant of -4.06 ± 0.05 (Estimate ± SE; t_119_ = 57.36, p<0.001) and an asymptotic value of 0.90 ± 0.02 (t_119_ = -75.81, p<0.001) or 90% active tussocks above 400 mm. With seasonal precipitation above 200 mm, 95% of the asymptotic value (equivalent to 86% active tussocks) was reached (Fig 1).

**Fig 2:**
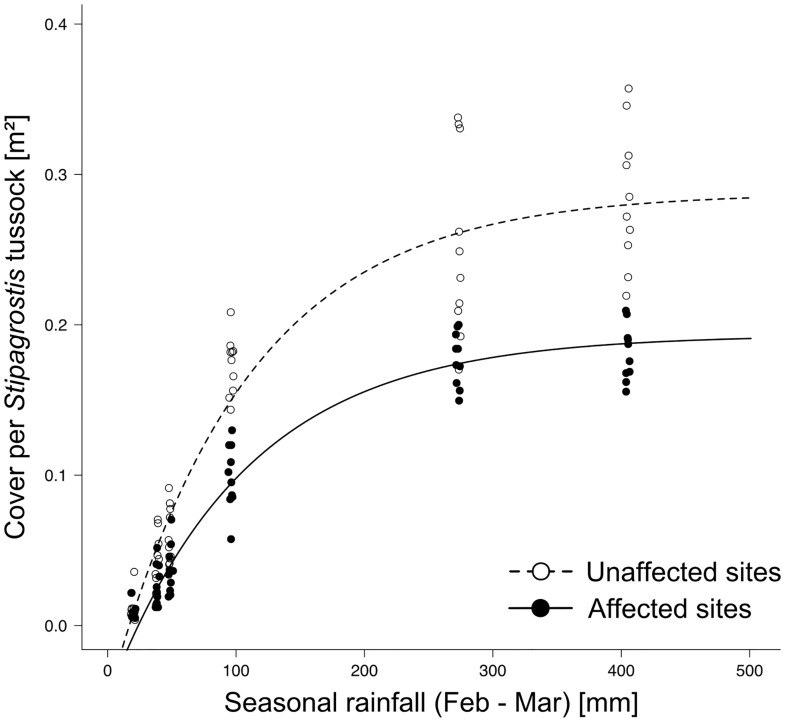
Cover per *Stipagrostis* tussock in relation to seasonal rainfall (February-March) on unaffected sites and affected sites between 2009 and 2016. Predicted lines from non-linear mixed effect models with asymptotic function are shown.

On both affected and unaffected sites, cover and hence biomass of *Stipagrostis* increased asymptotically with the amount of seasonal rainfall (Fig 2; Table A in S2 Table). In either case 95% of the asymptotic value was reached around 315 mm. *Crotalaria podocarpa* did not significantly influence the rate constant *lrc* nor the intercept of the x-axis *r0*, but on affected sites the maximum area per individual tussock (0.19 ± 0.01 m^2^) was significantly lower than on unaffected sites (0.29 ± 0.01 m^2^) (Estimate ± SE: 0.09 ± 0.13, t_97_ = 7.15, p<0.001). Grass productivity was continuously 30–35% less on affected compared to unaffected sites; higher seasonal rainfall did not compensate for this lower productivity.

**Fig 3:**
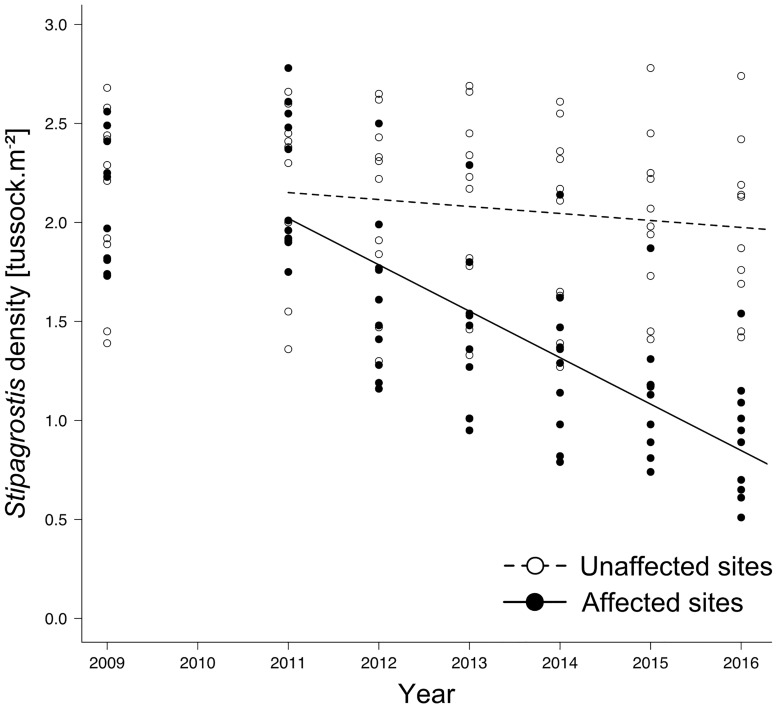
Trend of *Stipagrostis* tussock density on unaffected and affected sites between 2011 and 2016. Predicted lines for linear mixed effect models are shown.

On unaffected sites, the density of *Stipagrostis* tussocks remained largely at its initial level with 2.16 ± 0.14 tussocks/m^2^ and decreased only by -0.04 ± 0.01 (t_49_ = -4.87, p<0.001) per year throughout the observation period. In contrast, on affected sites, *Stipagrostis* density decreased steadily (Estimate ± SE: -0.23 ± 0.02, t_49_ = -14.30, p<0.001) from 2.02 ± 0.11 tussocks/m^2^ to 0.91± 0.09 in 2016, reducing tussock density to less than 45% (Fig 3).

**Fig 4:**
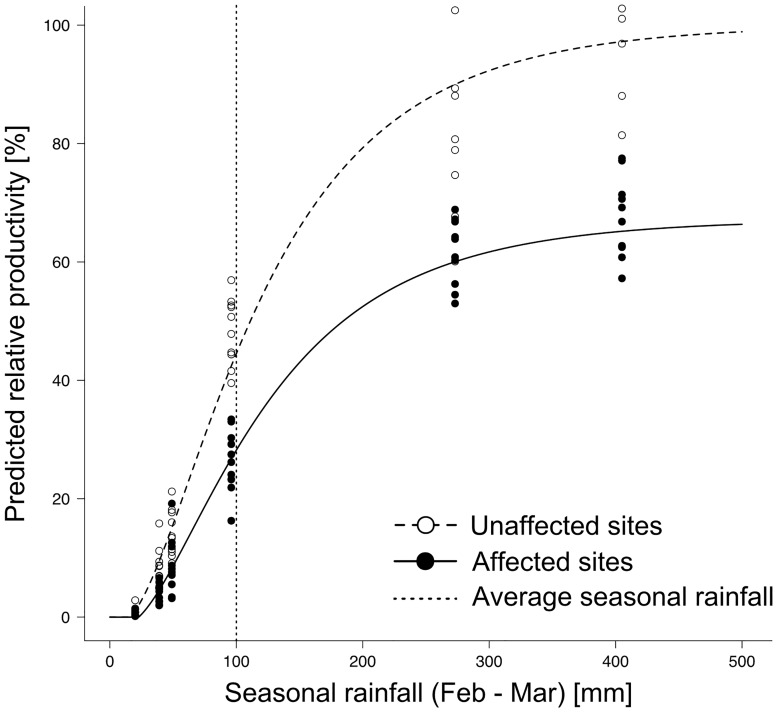
Predicted relative productivity of the land at a given tussock density, combined from the predicted values for tussock dormancy and individual tussock productivity in relation to seasonal rainfall (Feb-Mar) on unaffected and affected sites. Dots indicate measured values, vertical line marks average seasonal rainfall in the study region.

Projected relative land productivity (as combined index of the percentage of dormant tussocks and individual tussock) and hence relative carrying capacity is zero below ~24 mm seasonal rainfall. Above this threshold, a steep, quasi-linear increase up to 100 mm occurs, but later flattens out, reaching an asymptotic upper limit and 95% of potential capacity above 330 mm only (Fig 4). Similar to the individual production of tussocks, the relative carrying capacity on affected sites was continuously 30% less than on unaffected sites.

## Discussion

Our study provides evidence that the massive spread of the herbaceous legume *C*. *podocarpa* has a significant impact on *Stipagrostis* biomass production. The presence of high numbers of *C*. *podocarpa* did not affect the percentage of active grass tussocks, but decreased *Stipagrostis* productivity by 30% and, over time, reduced grass tussock density on the affected sites to less than 50% of its maximum. Both the percentage of active tussocks and *Stipagrostis* biomass production increased asymptotically with seasonal rainfall, but with additional rainfall there was no compensation of the reduced grass productivity in *Crotalaria*-affected areas. Rainfall had also no influence on grass tussock density.

### Effect of rainfall on grass productivity

As expected, the main factor driving the vegetation productivity of such arid rangelands is rainfall [21,47]. The production of grass biomass is thereby determined by both, the number of grass tussocks that are active following a certain amount of rainfall and the respective individual growth of the grass tussocks. Our models predict no measurable growth below 20 mm of seasonal rainfall, which conforms with Seely [36] and Henschel et al. [37] who researched grass biomass production along a rainfall gradient in the Namib desert. Both studies confirm a similar lower threshold for *Stipagrostis* productivity, but assume a linear relationship between biomass production and amount of rainfall. However, the rainfall covered in these studies ranged only between 0 and 100 mm, in which our results show quasi-linear behaviour too. Above 100 mm we found that productivity is clearly limited and exhibits an asymptotic behaviour and biomass production of individual tussocks approaches their maximum above ~315 mm. Additional rainfall does not substantially increase productivity. This suggests either a biological growth limit or a limiting resource availability other than water, such as nitrogen, as the soils in the study region are poor in nutrients [12,48,49]. Correspondingly, the potential land productivity and hence carrying capacity is also reached above 315 mm when both the percentage of active tussocks and individual productivity of tussocks are highest. Under the current rainfall regime in the study region, with an average of 100 mm seasonal rainfall, the carrying capacity of these lands reaches less than 50% of its potential. Even with 180 mm seasonal rainfall, only 75% of the potential carrying capacity is reached.

### Effect of *C*. *podocarpa* on grass productivity and density

The presence of *C*. *podocarpa* neither changed the general pattern of the *Stipagrostis* biomass–rainfall relationship nor affected the grasses dormancy state, but had an immediate and considerable negative impact on the productivity of *Stipagrostis* tussocks. This reduced growth of *Stipagrostis* on *Crotalaria*-affected sites was not compensated for by higher rainfall, indicating rather a competition for below- or above-ground resources such as nitrogen [12,49] or light [50,51] instead of the expected competition for water, as suggested by other studies [49,52]. Further, the reduced productivity obviously weakens the affected tussocks and reduces their ability to survive through the dry season [53], which explains the dramatically decreasing tussock density on *Crotalaria*-affected sites.

### Implication for affected rangelands

Disregarding its potential effects on local plant and animal communities [9,54], our findings show serious implications for *C*. *podocarpa* encroached arid rangelands and confirm the experiences and fears of local farmers. Taking into account both the reduced individual productivity of *Stipagrostis* and the continued decline of *Stipagrostis* tussock density over the last 8 years, *C*. *podocarpa* encroachment leads to considerable losses in pastoral production on the affected lands. Although our study is focused on only one legume species, and we do not know whether the massive spread of *C*. *podocarpa* will be transient, the reduced individual productivity of *Stipagrostis* and the continued decline of *Stipagrostis* tussock density over the last 8 years lead to considerable losses in pastoral production on the affected lands. Further, *C*. *podocarpa* has a considerable seed bank with more than 350 seeds/m^2^ ([40], T. C. Wagner and C. Fischer, unpublished data) due to the high rainfalls over the last ten years (own rainfall data, Table A in S3 Table), its seeds remain viable for many years (T. C. Wagner and C. Fischer, unpublished data) and predicted higher rainfalls for the coming decades [55], make a fast decline of the species in the study region rather unlikely. Therefore it will probably take decades for the affected land to recover its former productivity. A study by Milton & Dean [35] found that seven years after removing of grass tussocks, *Stipagrostis* tussocks reached only 34% of their former density. Studies by Seymour et al. [56] and Wiegand & Milton [57] suggest that the recovery from such a loss might even take decades. Moreover, the ability of legumes to fix nitrogen [31,58] and their better photosynthetic response to rising CO_2_-levels will further favour those C_3_ plants against perennial C_4_ grasses [26,31,59]. Therefore it is very likely that other herbaceous legume species will increasingly spread in arid grasslands and savannas, similarly impairing grass productivity. Consequently, increased attention to this phenomenon of herbaceous encroachment and the underlying processes seems advisable, to allow the development of timely and suitable management measures.

## Supporting Information

S1 Table Habitat characteristics of sites unaffected and affected by *Crotalaria podocarpa*. Differences in means tested by permutational t-tests (perm.t.test, n = 999), t and p values are given.
(DOCX)

S2 Table Fitted parameters for *Stipagrostis* tussock area. Parameter estimates ± SE for area per *Stipagrostis* tussock on unaffected and affected sites derived from non-linear mixed effect model with asymptotic function, *t*-values with 97 degrees of freedom.
(DOCX)

S3 Table Annual and seasonal rainfall on Rooiklip between 2001 and 2016. Rainfall recorded at the rain gauge near the Rooiklip farmhouse on a daily basis.
(DOCX)
