# Supplementary material for: Herbaceous Legume Encroachment Reduces Grass Productivity and Density in Arid Rangelands
Source: PLoS One. 2016 Nov 17;11(11):e0166743. doi: 10.1371/journal.pone.0166743 (PMC5113976; doi:10.1371/journal.pone.0166743)
Supplement: S1 Table — Differences in means tested by permutational t-tests (perm.t.test, n = 999), t and p values are given. (DOCX) [file pone.0166743.s001.docx]

**S1 Table A. Habitat characteristics of sites unaffected and affected by *Crotalaria podocarpa*.** Differences in means tested by permutational t-tests (perm.t.test, n=999), t- and p-values are given.

| Habitat characteristics | Unaffected | | | Affected | | | Unaffected vs. affected | |
| --- | --- | --- | --- | --- | --- | --- | --- | --- |
|  | Mean ± SE | Min | Max | Mean ± SE | Min | Max | t | p |
| Soil structure | | | | | | | | |
| Sand | 22.5 ± 1.3 | 15 | 30 | 24.0 ± 1.6 | 15 | 30 | 0.71 | 0.64 |
| Pebbles | 67.5 ± 1.5 | 60 | 75 | 65.5 ± 1.7 | 60 | 75 | -0.86 | 0.49 |
| Medium Stones | 2.2 ± 0.4 | 1 | 5 | 2.4 ± 0.3 | 1 | 5 | 0.37 | 1.00 |
| Large Stones | 1.6 ± 0.4 | 0 | 3 | 1.6 ±0.4 | 0 | 3 | 0.00 | 1.00 |
| Rock | 4.9 ± 0.6 | 3 | 10 | 4.6 ± 1.0 | 1 | 10 | -0.25 | 0.86 |
| Vegetation structure | | | | | | | | |
| Shrubs | 0.9 ± 0.4 | 0 | 3 | 1.0 ± 0.5 | 0 | 5 | 0.17 | 1.00 |
| Trees | 0.0 ± 0.0 | 0 | 0 | 0.0 ± 0.0 | 0 | 0 | ? | 1.00 |
| Initial density *C. podocarpa* | 3.0 ± 0.7 | 0 | 7 | 76.4 ± 12.3 | 24 | 166 | 5.96 | 0.001 |
| Initial density *Stipagrostis* | 212.7 ± 14.3 | 139 | 268 | 210.1 ± 10.2 | 173 | 256 | -0.15 | 0.89 |
