# Supplementary material for: Herbaceous Legume Encroachment Reduces Grass Productivity and Density in Arid Rangelands
Source: PLoS One. 2016 Nov 17;11(11):e0166743. doi: 10.1371/journal.pone.0166743 (PMC5113976; doi:10.1371/journal.pone.0166743)
Supplement: S2 Table — Parameter estimates ± SE for area per Stipagrostis tussock on unaffected and affected sites derived from non-linear mixed effect model with asymptotic function, t-values with 97 degrees of freedom. (DOCX) [file pone.0166743.s002.docx]

**S2 Table A. Fitted parameters for *Stipagrostis* tussock area.** Parameter estimates ± SE for area per *Stipagrostis* tussock on unaffected and affected sites derived from non-linear mixed effects model with asymptotic function, *t*-values with 97 degrees of freedom

|  | Unaffected sites | | | Affected sites | | |
| --- | --- | --- | --- | --- | --- | --- |
|  | Estimate ± SE | *t* | *p* | Estimate ± SE | *t* | *p* |
| Asymp | 0.29 ± 0.01 | 26.70 | < 0.001 | 0.19 ± 0.01 | 19.55 | < 0.001 |
| RO | -0.05 ± 0.01 | -5.78 | < 0.001 | -0.05 ± 0.01 | -5.78 | < 0.001 |
| lrc | -4.68 ± 0.08 | -56.12 | < 0.001 | -4.68 ± 0.08 | -56.77 | < 0.001 |
