# Supplementary material for: Herbaceous Legume Encroachment Reduces Grass Productivity and Density in Arid Rangelands
Source: PLoS One. 2016 Nov 17;11(11):e0166743. doi: 10.1371/journal.pone.0166743 (PMC5113976; doi:10.1371/journal.pone.0166743)
Supplement: S3 Table — Rainfall recorded at the rain gauge near the Rooiklip farmhouse on a daily basis. (DOCX) [file pone.0166743.s003.docx]

**S3 Table A. Annual and seasonal rainfall on Rooiklip between 2001 and 2016.** Rainfall recorded at the rain gauge near the Rooiklip farmhouse on a daily basis. Mean annual precipitation is 120 mm, provided by Namibia Meteorological Service.

| Year | Annual Rainfall [mm] | Rainfall Feb-Mar [mm] | Number of rainy days | Number of discrete Rainfall events | Number of rainy periods > 1d | Average length of humid periods [d] |
| --- | --- | --- | --- | --- | --- | --- |
| 2001 | 106 | 10 | 13 | 9 | 2 | 1.44 |
| 2002 | 115 | 90 | 22 | 11 | 6 | 1.57 |
| 2003 | 111 | 45 | 17 | 12 | 4 | 1.33 |
| 2004 | 118 | 34 | 21 | 15 | 5 | 1.43 |
| 2005 | 54 | 35 | 7 | 5 | 1 | 1.40 |
| 2006 | 342 | 104 | 32 | 16 | 8 | 2.00 |
| 2007 | 27 | 5 | 3 | 3 | 0 | 1.00 |
| 2008 | 279 | 127 | 43 | 20 | 9 | 2.25 |
| 2009 | 290 | 273 | 24 | 15 | 5 | 1.60 |
| 2010 | 134 | 56 | 16 | 13 | 3 | 1.31 |
| 2011 | 619 | 405 | 50 | 25 | 15 | 2.10 |
| 2012 | 108 | 96 | 16 | 12 | 3 | 1.33 |
| 2013 | 187 | 20 | 18 | 11 | 3 | 1.64 |
| 2014 | 198 | 162 | 23 | 12 | 6 | 1.77 |
| 2015 | 101 | 39 | 14 | 10 | 2 | 1.40 |
| 2016 | - | 49 | 12* | 8* | 4* | 1.80* |
| **Average 2001-2015** | **179** | **101** | **23** | **13** | **5** | **1.59** |

* only Jan – Oct available
